# The role of acupuncture in the treatment of women with pain in endometriosis: A protocol for systematic review and meta-analysis

**DOI:** 10.1097/MD.0000000000027582

**Published:** 2021-12-10

**Authors:** Qin Yan, Jing Li, Jing Zeng

**Affiliations:** a Department of Urology, Jingzhou Traditional Chinese Medicine Hospital of Hubei Province, Jingzhou , China; b Department of Pediatrics, Jingzhou Traditional Chinese Medicine Hospital of Hubei Province, Jingzhou, China; c Department of Gynecology, Jingzhou Traditional Chinese Medicine Hospital of Hubei Province, Jingzhou, China.

**Keywords:** acupuncture, endometriosis, pain treatment, pelvic pain

## Abstract

**Background:**

Given the high numbers of recent cases related to Endometriosis, acupuncture has become a first line of treatment to alleviate the discomfort caused by endometriosis. Numerous studies have reported that acupuncture has a distinct effect when treating the discomfort caused by endometriosis. The primary advantages include various treatment methods, simple administration, minimal adverse reactions, and having no impact on the intrauterine environment. This study aims to elucidate the role of acupuncture in treating pain associated with endometriosis.

**Methods:**

The authors will search 6 online-based databases to find Randomized Controlled Trials related to determining the role of acupuncture in treating pain from endometriosis. The assessed primary outcomes include the clinical effective rate, variation in the level of pain, and variation in peripheral blood CA-125 level. A comprehensive meta-analysis statistical software will be used to conduct all analyses.

**Results:**

This study will assess the role of acupuncture when it is used to treat pain arising from endometriosis.

**Conclusion:**

The conclusions presented in the metanalysis will present a scientific-based theoretical framework and a standardized clinical guidance for treating endometriosis-related pain.

**Ethics and dissemination:**

This systematic review and meta-analysis does not require an ethics approval as it does not collect any primary data from patients.

**OSF registration number:**

September 28, 2021.osf.io/htukv. (https://osf.io/htukv/).

## 1 Introduction

Endometriosis (EM) is a condition where endometrial tissue growth appears in parts outside the uterine cavity and myometrium, forming nodules and masses, causing clinical symptoms, such as pelvic pain and infertility.^[1,2]^ Endometriosis is common among adult females of childbearing age. As a result, the disease has attracted extensive attention in the medical community. Among its symptoms, recurrent pain lowers the overall wellbeing of women. As a result, EM directly affects patients’ physiological, psychological, and social behavior. Despite its high incidence, the clinical pathogenesis of EM is not clear. Relevant studies indicate that the occurrence of EM is related to psychological cognition, estrogen, central nervous system, inflammatory response nerve fiber injury, and regeneration are closely related.^[3,4]^ Endometriosis has a long clinical treatment cycle, and it is difficult to completely cure the disease.^[5]^ So far, the etiology of EM and its pain mechanism have not been clearly studied. As a result, it is difficult to clinically deal with symptoms associated with EM, such as pelvic pain. As a result, EM has a serious impact on the work and life of sick women. Throughout the entire course of clinically treating this disease, treating the pain due to endometriosis remains a crucial link in managing the disease. Detailed analysis of the pathogenic mechanism and effective treatment scheme of endometriosis pain can fundamentally improve the overall wellbeing of endometriosis patients. Admittedly, acupuncture is commonly used in the treatment of long-term pain. However, the results are still contentious.^[6]^ This study will provide a scientific theoretical basis and standardized clinical guidance for treating pain in endometriosis patients. It will improve the efficacy of using acupuncture to treat EM.

## 2 Materials and methods

The protocol will be presented according to the Preferred Reporting Items for Systematic Reviews and Meta-Analyses for Protocols guidelines.^[7,8]^ The authors will conduct a search for related studies in 6 online-based databases, including, Web of Science, MEDLINE, PubMed, WanFang database, Cochrane Library, Embase, and China National of Knowledge Infrastructure.

### 2.1 Search strategy

A pair of reviewers will perform an independent screening of all eligible studies. Afterward, there will be a screening process of the titles, keywords, and abstracts. Afterward, the irrelevant studies will be excluded. In the case where the full-text literature is irretrievable, the researcher needs to contact the author. The reviewer will then scrutinize the article to determine whether it could be included and record the reasons for excluding the study. In the case where multiple studies report data of a particular trial, the authors will include the most recent study or the article that studies a larger sample size. Lastly, all disagreements between the first 2 reviewers will seek a third independent reviewer to resolve the disagreement.

### 2.2 Data selection and analysis

NoteExpress 3.0 document manager will be used to extract data and to classify and sort documents in the literature quality evaluation. An improved Jadad scale will be adopted to assess the quality of each study, the evaluation will be done from the aspects of random sequence generation method, blind method, randomized hiding, withdrawal, and withdrawal (1–3 points will be regarded as low quality and 4–7 points will be considered as high quality). Data extraction and quality evaluation are carried out by the 2 researchers through parallel discussions. If the results are inconsistent, the agreement reached by the 2 reviewers through discussion will be adopted.

### 2.3 Statistical analysis method

All statistical analysis relating to the included studies will be done with Revman5.3 software. The Relative Risk will be adopted to count the effect quantity. Meanwhile, the weighted mean difference will be used to measure the data. Both are considered statistically significant with 95% confidence intervals, *P* < .05. Based on the results of the heterogeneity test, a fixed-effects model (*P* > .1 or *I*^*2*^ ≤ 50%) or a random effects model (*P* < .1 or *I*^*2*^ ≥ 50%) will be used for meta-analysis.

### 2.4 Assessment of publication biases

This study will also conduct a comprehensive protocol review of all the eligible studies to assess any reporting bias. A funnel plot will be used to assess publication bias if the number of included studies goes beyond 10.

### 2.5 Outcomes

Clinical effective rate, differences in the main level of pain, differences in the peripheral blood CA-125 levels.

A dichotomous analysis of the overall effectiveness of using acupuncture therapy will be done subjectively. Accordingly, the percentage of participants who experience relief from the pain caused by endometriosis following the acupuncture treatment will be indicated by the responses of the patients to the evaluation criteria. The primary treatment outcome measurement involves any change in the pelvic pain level not related to menses or sexual activity. The study will use the Endometriosis Symptom Severity Scale.^[9]^ The levels of blood CA-125 were determined in the pre- and-posttreatment periods with an enzyme-linked immunosorbent assay.^[10]^

## 3 Discussion

Generally, endometriosis presents severe pain of various origins. According to previous studies, it is reasonable to have confidence in using acupuncture to reduce the pain in some patients. As an analgesic form of therapy, the effect of acupuncture and moxibustion has been considered as an overall safe alternative therapy in various studies, with few side effects (harmless) and without any environmental impact. Therefore, it could also be considered as a “sustainable” treatment form. This study provides a reference basis for acupuncture and moxibustion in treating pain arising from endometriosis.

## Author contributions

**Conceptualization:** Qin Yan, Jing Li, Jing Zeng.

**Data curation:** Qin Yan, Jing Li, Jing Zeng.

**Formal analysis:** Qin Yan, Jing Li, Jing Zeng.

**Funding acquisition:** Qin Yan, Jing Li, Jing Zeng.

**Investigation:** Qin Yan, Jing Li, Jing Zeng.

**Methodology:** Jing Li, Jing Zeng.

**Project administration:** Jing Li.

**Resources:** Qin Yan, Jing Li, Jing Zeng.

**Software:** Qin Yan, Jing Zeng.

**Validation:** Qin Yan, Jing Li, Jing Zeng.

**Visualization:** Qin Yan, Jing Li, Jing Zeng.

**Writing – original draft:** Qin Yan, Jing Li.

**Writing – review & editing:** Qin Yan, Jing Li, Jing Zeng.
